# Validation of Prediction Rules for Computed Tomography Use in Children With Blunt Abdominal or Blunt Head Trauma: Protocol for a Prospective Multicenter Observational Cohort Study

**DOI:** 10.2196/43027

**Published:** 2022-11-24

**Authors:** Irma T Ugalde, Pradip P Chaudhari, Mohamed Badawy, Paul Ishimine, Kevan A McCarten-Gibbs, Kenneth Yen, Nisa S Atigapramoj, Allyson Sage, Donovan Nielsen, P David Adelson, Jeffrey Upperman, Daniel Tancredi, Nathan Kuppermann, James F Holmes

**Affiliations:** 1 Department of Emergency Medicine Children's Memorial Hermann Hospital McGovern Medical School at UTHealth Houston Houston, TX United States; 2 Division of Emergency and Transport Medicine Children's Hospital Los Angeles Keck School of Medicine of the University of Southern California Los Angeles, CA United States; 3 Department of Pediatrics University of Texas Southwestern Dallas, TX United States; 4 Department of Emergency Medicine and Pediatrics University of California San Diego School of Medicine Rady Children's Hospital San Diego, CA United States; 5 Department of Emergency Medicine University of California San Francisco Benioff Children's Hospital Oakland, CA United States; 6 Department of Emergency Medicine University of California Davis School of Medicine Sacramento, CA United States; 7 Barrow Neurological Institute of Phoenix Children's Hospital Department of Child Health, Division of Pediatric Neurosurgery University of Arizona College of Medicine Phoenix, AZ United States; 8 Department of Pediatric Surgery Monroe Carell Jr. Children's Hospital Vanderbilt University School of Medicine Nashville, TN United States; 9 Department of Pediatrics University of California Davis School of Medicine Sacramento, CA United States

**Keywords:** pediatric trauma, intra-abdominal injury, traumatic brain injury, clinical prediction rules, emergency medicine

## Abstract

**Background:**

Traumatic brain injuries (TBIs) and intra-abdominal injuries (IAIs) are 2 leading causes of traumatic death and disability in children. To avoid missed or delayed diagnoses leading to increased morbidity, computed tomography (CT) is used liberally. However, the overuse of CT leads to inefficient care and radiation-induced malignancies. Therefore, to maximize precision and minimize the overuse of CT, the Pediatric Emergency Care Applied Research Network (PECARN) previously derived clinical prediction rules for identifying children at high risk and very low risk for IAIs undergoing acute intervention and clinically important TBIs after blunt trauma in large cohorts of children who are injured.

**Objective:**

This study aimed to validate the IAI and age-based TBI clinical prediction rules for identifying children at high risk and very low risk for IAIs undergoing acute intervention and clinically important TBIs after blunt trauma.

**Methods:**

This was a prospective 6-center observational study of children aged <18 years with blunt torso or head trauma. Consistent with the original derivation studies, enrolled children underwent routine history and physical examinations, and the treating clinicians completed case report forms prior to knowledge of CT results (if performed). Medical records were reviewed to determine clinical courses and outcomes for all patients, and for those who were discharged from the emergency department, a follow-up survey via a telephone call or SMS text message was performed to identify any patients with missed IAIs or TBIs. The primary outcomes were IAI undergoing acute intervention (therapeutic laparotomy, angiographic embolization, blood transfusion, or intravenous fluid for ≥2 days for pancreatic or gastrointestinal injuries) and clinically important TBI (death from TBI, neurosurgical procedure, intubation for >24 hours for TBI, or hospital admission of ≥2 nights due to a TBI on CT). Prediction rule accuracy was assessed by measuring rule classification performance, using standard point and 95% CI estimates of the operational characteristics of each prediction rule (sensitivity, specificity, positive and negative predictive values, and diagnostic likelihood ratios).

**Results:**

The project was funded in 2016, and enrollment was completed on September 1, 2021. Data analyses are expected to be completed by December 2022, and the primary study results are expected to be submitted for publication in 2023.

**Conclusions:**

This study will attempt to validate previously derived clinical prediction rules to accurately identify children at high and very low risk for clinically important IAIs and TBIs. Assuming successful validation, widespread implementation is then indicated, which will optimize the care of children who are injured by better aligning CT use with need.

**International Registered Report Identifier (IRRID):**

RR1-10.2196/43027

## Introduction

### Background

Traumatic brain injuries (TBIs) and intra-abdominal injuries (IAIs) are leading causes of death and disability in children aged >1 year [1]. More than 600,000 children who are injured are evaluated annually for IAI in US emergency departments (EDs), and approximately 25% undergo abdominal computed tomography (CT) imaging [2-4]. However, 90% of children undergoing abdominal CT do not have IAIs [5-7]. In addition, more than 650,000 children with blunt head trauma are evaluated for potential TBIs annually in US EDs; of these children, approximately 50% undergo cranial CT scans [2-4]. Fewer than 10% of imaged children, however, have TBIs on CT, which suggests the inefficient use of this technology [8,9].

For more than 2 decades, CT scanning has been the diagnostic imaging method of choice to detect IAIs and TBIs in children [10,11]. CT is highly accurate in diagnosing IAIs and TBIs, which decreases the level of clinical monitoring required and is an important factor in determining the need for surgical treatment [12,13]. CT scanning also presents risks to children from ionizing radiation [2,4,14-24]. The lifetime attributable risk of a solid cancer from 1 abdominal CT scan is estimated to be as high as 1 per 485 abdominal CT scans for children, whereas the risk of a solid cancer from cranial CT scans in children is estimated to be as high as 1 in 960 [4]. The appropriate use of CT, targeted to the population of children who are injured who would benefit from the test, is an area for quality and safety improvement.

Several professional societies and organizations have called for action to promote the judicious use of imaging in patient care [25], including recommendations to (1) perform only necessary CT examinations, (2) encourage the development and adoption of pediatric CT protocols, and (3) encourage the use of selective strategies for pediatric imaging. Our study addresses these issues directly by proposing to validate, in a large, diverse population, previously derived clinical prediction rules for children with blunt abdominal or head trauma.

Clinical prediction rule development has become an important research area aimed at helping clinicians optimize the decision-making process at the point of patient care [26-28]. ED clinicians support the development of prediction rules for determining the use of radiographic imaging after traumatic injuries [29,30]. Previously, clinical prediction rules to identify children with abdominal or minor head trauma (presenting Glasgow Coma Scale scores of 14-15) at high and very low risk of IAI undergoing acute intervention and clinically important TBI were developed in the Pediatric Emergency Care Applied Research Network (PECARN), with the goal of optimizing CT use [7,9]. However, a critical piece to rule implementation is multicenter validation [26], as the spectrum and evaluation of traumatic injuries varies between centers [31]. In the hierarchy of creation to implementation, prediction rules that have been derived but not validated are the lowest level of evidence (Level 4), and rules that have been broadly validated at multiple sites advance to Level 2 [26]. In this study, we aimed to externally validate these prediction rules and raise the level of evidence so that implementing the rules in clinical practice in all EDs is appropriate and subsequent impact studies can be performed.

### Objectives

The objectives of the current study were to (1) validate the previously derived PECARN clinical prediction rule that accurately and precisely identifies children at near zero risk of IAIs undergoing acute intervention; (2) validate the previously derived PECARN clinical prediction rules that accurately and precisely identify children aged <2 years and those aged 2-18 years at near zero risk of clinically important TBIs; and (3) identify factors associated with CT use in children considered very low risk for IAIs or TBIs by the prediction rules. For the validation of both prediction rules, we planned the sample size to have sufficient power to ensure very narrow CIs around point estimates for the sensitivities and negative predictive values (NPVs) of the prediction rules.

## Methods

### Study Overview

This was a prospective, multicenter observational study of children aged <18 years who presented to the ED with blunt torso trauma or nontrivial blunt head trauma. The study was endorsed by the Clinical Translational Science Award Emergency Care Translational Research Collaborative and was conducted at 6 of the participating hospitals in the network. The 6 sites are dedicated pediatric EDs with high volumes of pediatric trauma; 4 are in California and 2 in Texas. The goal was to validate previously derived and highly accurate prediction rules for IAI undergoing acute intervention and clinically important TBI in children.

### Study Population

#### Inclusion and Exclusion Criteria

Children aged <18 years with blunt abdominal trauma, head trauma, or both presenting to the participating EDs were enrolled based on the same inclusion and exclusion criteria as the derivation studies. The specific criteria are listed in Textboxes 1 and 2 [7,9]. Patients with both blunt head and abdominal trauma were entered into *both* cohorts, as in both prior PECARN derivation studies [7,9]. In addition, we enrolled all eligible children whether or not CT scans were obtained. Due to ethical concerns, the use of CT scans was not mandated by the study protocol, but all patients were followed to detect outcomes, including SMS text messaging or telephone follow-up of those discharged home from the ED.

**Intra-abdominal injury (IAI) cohort inclusion and exclusion criteria.**

**Inclusion criteria**

1. Aged <18 years
2. Blunt torso trauma resulting from a substantial mechanism of injury defined as [32,33]:
  Motor vehicle collision >60 mph, ejection, or rollover
  Automobile versus pedestrian or bicycle with automobile speed >25 mph
  Falls >20 feet in height
  Crush injury to the torso
  Physical assault involving the abdomen
3. Decreased level of consciousness (Glasgow Coma Scale score <15) in association with blunt torso trauma
4. Blunt traumatic event, regardless of the mechanism, with either extremity paralysis or multiple long bone fractures at multiple sites (eg, tibia and humerus fracture)
5. History and physical examination suggestive of IAI following blunt torso trauma of any mechanism (including mechanisms of injury of less severity than mentioned above), including any abdominal imaging (computed tomography or focused assessment with sonography for trauma), chest and pelvis radiographs, or laboratory screening for IAI

**Exclusion criteria**

1. Penetrating trauma
2. Preexisting neurological disorders seriously confounding physical examination assessment (eg, profound mental retardation or cerebral palsy)
3. Traumatic injury occurring more than 24 hours prior to the time of presentation to the emergency department
4. Transfer of the patient to the participating center from an outside facility with prior abdominal computed tomography imaging
5. Strong suspicion that the injury was the result of child abuse (eg, skeletal survey ordered)
6. Known pregnancy
7. Prisoner

**Traumatic brain injury (TBI) cohort inclusion and exclusion criteria.**

**Inclusion criteria**

1. Aged <18 years
2. Nontrivial minor blunt head trauma (defined by Glasgow Coma Scale [GCS] scores of 14-15 at emergency department presentation)
  - Cranial computed tomography performed following trauma were assumed to have nontrivial minor blunt head trauma for the purposes of inclusion in this cohort.

**Exclusion criteria**

1. Children with trivial mechanisms of injury defined by falls from ground level, or walking or running into stationary objects, *and* the lack of any signs or symptoms of head trauma or the presence of scalp lacerations or abrasions alone
2. GCS score <14, except for those who had a posttraumatic seizure and the postrecovery GCS score was 14-15
3. Penetrating trauma (eg, gunshot or knife wounds)
4. Preexisting neurological disorders seriously confounding physical examination assessment (eg, substantial mental retardation or cerebral palsy)
5. Preexisting ventriculo-peritoneal or ventriculo-atrial shunts (or similar devices)
6. Traumatic injury occurring more than 24 hours prior to the time of presentation to the emergency department
7. Transfer of the patient to the participating center with prior cranial computed tomography or magnetic resonance imaging
8. Preexisting brain tumor or history of a bleeding disorder (eg, hemophilia and von Willebrand disease)
9. Strong suspicion that the injury was the result of child abuse (eg, skeletal survey ordered)
10. Prisoner

#### Clinician Survey Inclusion Criteria

A survey of the clinicians providing initial evaluation and care to eligible patients at each participating site was conducted prior to study participation (see below). In this survey, we queried clinicians about perceptions and the frequency of use of prediction rules. As new faculty or fellow clinicians were hired at participating sites, they received the survey prior to study participation.

### Study Procedures

#### ED Data Collection

Children enrolled in the study underwent history and physical examinations per standard clinical care. The emergency clinician providing care (eg, emergency medicine attending physician, pediatric emergency medicine attending clinician, or trauma surgeon) was prompted to complete the standardized case report form (Multimedia Appendix 1) *prior* to knowing the results of diagnostic testing or imaging results (if performed) to avoid observation bias. This clinician then collected and recorded the limited number of variables that constituted each prediction rule (Textbox 3) [7,9]. In addition, clinicians self-reported their unstructured suspicion for the risk of IAI or TBI for all patients and the indications for imaging in those who underwent CT imaging. Only one case report form was completed for each patient.

**Prediction rule variables for intra-abdominal injury and traumatic brain injury.**

**Intra-abdominal Injury Prediction Rule Variables**

- Does the patient complain of abdominal pain?
- Has the patient vomited since the time of injury?
- Is the patient’s Glasgow Coma Scale (GCS) score <14?
- Does the patient have absent or decreased breath sounds?
- Does the patient have any thoracic wall trauma? (eg, erythema, abrasions, ecchymosis, subcutaneous air, or laceration)
- Does the patient have any abdominal wall trauma? (eg, seatbelt sign, erythema, abrasions, ecchymosis, subcutaneous air, or laceration)
- Does the patient have abdominal tenderness?

**Traumatic Brain Injury Prediction Rule Variables, Aged <2 Years**

- Does the patient have a GCS score <15 or altered mental status? (eg, slow to respond, agitation, sleepiness, confusion, or repetitive questioning)
- Does the patient have a nonfrontal scalp hematoma?
- Was there a loss of consciousness ≥5 seconds?
- Does the patient have a palpable skull fracture or is it unclear due to scalp swelling?
- Is the patient acting abnormally per the parent or guardian?
- Was there a severe mechanism of injury? (defined as motor vehicle crash with patient ejection, death of another passenger, or rollover; pedestrian or bicyclist without helmet struck by a motorized vehicle; fall >3 feet; or head struck by a high-impact object [substantially heavy object struck head, baseball, horse kick, etc])

**Traumatic Brain Injury Prediction Rule Variables, Aged 2-18 Years**

- Does the patient have a GCS score <15 or altered mental status? (eg, slow to respond, agitation, sleepiness, confusion, or repetitive questioning)
- Was there a loss of consciousness?
- Has the patient vomited since the injury?
- Are there clinical signs of basilar skull fracture?
- Does the patient have a severe headache (score of 8 or greater out of 10)?
- Was there a severe mechanism of injury? (defined as motor vehicle crash with patient ejection, death of another passenger, or rollover; pedestrian or bicyclist without helmet struck by a motorized vehicle; fall >5 feet; or head struck by a high-impact object [substantially heavy object struck head, baseball, horse kick, etc])

For the TBI cohort, the rule variables were different for those aged <2 years versus those aged ≥2 years, as per the derivation study [9]. For the IAI cohort, complaints on patient history that required verbal skills (eg, abdominal pain) were recorded as “preverbal” for children aged <3 years. Completed case report forms were entered by the site research coordinator into Research Electronic Data Capture (REDCap; Vanderbilt University), a secure, Health Insurance Portability and Accountability Act (HIPAA)–compliant web application for building databases and managing web-based data for research [34].

Diagnostic imaging was performed at the discretion of the emergency clinician caring for the patient, with instructions to complete the case report form before knowledge of CT imaging results. In the uncommon event that patient instability or other issues precluded recording study data prior to knowledge of the imaging results, the clinician was instructed to complete the case report form once the patient was stabilized. Based on our previous studies and the Glasgow Coma Scale eligibility criteria, we had anticipated that this scenario would occur infrequently. At a random interval and for a limited number of enrollments (approximately 250 enrollments for both the TBI and IAI cohorts, for a total of 500 enrollments), the study team collected whether any CT imaging was reviewed prior to completing study-related ED case report forms.

Diagnostic radiologic imaging examinations were performed according to each site’s radiology protocol. Final interpretations of all CT and magnetic resonance imaging scans as well as angiographic studies were made by attending radiologists at each study site. With site principal investigator oversight, research coordinators identified all abdominal and cranial CT scans with any traumatic or nontraumatic abnormalities. The dictated CT scan reports of these abnormal CT scans were entered into the electronic database. Detailed data from these abnormal abdominal or cranial CT scans were then further abstracted by trained abstractors. Dictated reports for all magnetic resonance imaging and angiographic studies, regardless of the presence or absence of any abnormalities, were additionally uploaded into the database. For patients with radiologic reports containing the wording “questionable” or “possible,” we relied on the treating clinicians’ determinations.

#### Patient Follow-up Procedures

The guardians of enrolled patients were provided an information sheet about the study. This information sheet notified the guardians of patients discharged from the ED that they would be contacted by telephone or SMS text messaging by the research coordinator from 1 week to 3 months after the initial ED visit. The research coordinator followed scripted survey instructions to document any possible missed IAIs that underwent acute intervention or clinically important TBIs. If an SMS text response was received that indicated the patient had a subsequent visit with a clinician related to the ED visit–associated abdominal or head injury, the research coordinator attempted to reach them by telephone or text to complete the additional questions on the survey.

When unable to contact the patient’s guardian after 4 telephone or SMS texting attempts extending to 3 months after the initial ED visit, we proceeded with alternative follow-up measures. We reviewed the patients’ medical records to ensure that no patients discharged from the ED and in whom telephone follow-up attempts had failed were subsequently diagnosed with an IAI, a TBI, or died in the weeks after the ED visit. If such an event was discovered, we collected all data involving the missed injury or death and had the etiology of the outcome adjudicated by a 3-member panel blinded to the ED presentation.

For patients who were hospitalized, data from the medical record were collected to determine outcome status. No telephone or SMS text messaging contact was attempted for these patients. We recorded the results of all imaging studies of interest obtained during the hospitalization. Patients were hospitalized at the discretion of the clinician(s) providing initial care, according to the standard practices of each site.

#### Missed Eligible Patients

Based on our previous experience [7,9], we had anticipated that approximately 80% of eligible patients would be enrolled; however, some eligible patients would inadvertently be missed. These patients were identified by electronic ED patient log review, and basic information about these missed eligible patients was documented on a separate case report form to allow for general comparisons between enrolled and missed patients and potential enrollment bias.

#### Survey of ED Clinicians

We surveyed the clinicians providing care at each study site prior to and during the enrollment of the study. This survey was performed to evaluate clinicians’ assessment of how well clinical predication rules perform, their knowledge of the rules, and how frequently they implemented these rules in practice. In this survey, we also collected basic information on clinician demographics, clinical experience, and current board eligibility or board certification. Responses were collected through an institutional review board (IRB)–approved survey accessed via a unique link in a scripted email notice sent out to each clinician through a REDCap database. Survey results were assigned an identification number unique to each clinician and entered into each case report form the clinician completed. Records linking the identification numbers and the clinician names were maintained by the research coordinator at the respective participating institutions to protect the clinician’s privacy and identifying information from the site investigators. In this way, we evaluated clinician characteristics associated with patients who had no PECARN risk factors for IAI undergoing acute intervention or clinically important TBI but who nonetheless underwent CT imaging.

### Study Outcomes

#### IAI Cohort Outcome Measure

Our primary outcome for the IAI cohort was IAI undergoing acute intervention [7]. An IAI was defined as any injury to the spleen, liver, urinary tract (kidney to bladder), pancreas, gastrointestinal tract (stomach to sigmoid colon including the mesentery), gallbladder, adrenal gland, or vascular structure or fascial defect (traumatic abdominal wall hernia). IAI undergoing acute intervention was defined as an IAI with any of the following: (1) therapeutic intervention at laparotomy (ie, necessary abdominal surgery); (2) angiographic embolization of a bleeding abdominal organ or other abdominal vascular structure; (3) blood transfusion for anemia due to intra-abdominal hemorrhage from an IAI; (4) administration of intravenous fluids for ≥2 nights to maintain hydration in patients unable to eat or drink in patients with pancreatic or gastrointestinal injuries; or (5) death due to the IAI.

#### TBI Cohort Outcome Measure

Our primary outcome for the TBI cohort was clinically important TBI [9]. TBI was defined as any extra-axial hematoma (subdural or epidural); subarachnoid hemorrhage; intraventricular hemorrhage; intraparenchymal hemorrhage or contusion; cerebral contusion, hemorrhage, or hematoma; cerebral edema; traumatic infarction; midline shift; herniation; venous sinus thrombosis; pneumocephalus; skull diastasis; shear injury; subpial hemorrhage; or skull fracture depressed by at least the table width of the skull. Clinically important TBI was defined as a TBI with any of the following: (1) neurosurgery for treatment of the TBI; (2) endotracheal intubation >24 hours for the TBI; (3) hospitalization of ≥2 nights for the head injury in association with TBI on CT; or (4) death due to the TBI.

### Statistical Analysis Plan

#### Statistical Analysis

We conducted separate analyses for the following specific aims: (1) validation of the IAI prediction rule; (2) validation of the 2 age-specific TBI prediction rules; and (3) measurement of interrater agreement of each of the clinical prediction rules. We assessed the accuracy of the rules by measuring the rule classification performance, using standard point and CI estimates of the operational characteristics of each prediction rule (sensitivity, specificity, positive predictive values, NPV, and diagnostic likelihood ratios).

For the IAI cohort, we aimed to validate the previously derived clinical prediction rule that accurately and precisely identifies children at near zero risk of IAIs undergoing acute intervention. The NPV of this rule must be nearly 100%, and sensitivity greater than 95%. For the TBI cohort, we aimed to validate the previously derived age-stratified clinical prediction rule that accurately and precisely identifies children at near zero risk of clinically important TBIs. The NPV of these rules must be nearly 100%, and sensitivity above 95%.

The statistical analyses and sample size requirements for the prediction rules were similar. Comparisons of specific characteristics (eg, age, sex, rate of IAI undergoing acute intervention, rate of clinically important TBI) of enrollees to nonenrollees were performed to detect potential enrollment biases.

#### Sample Size Estimates

Considering the consequences of false negative results, we specified that the validation of each prediction rule would need to meet 2 requirements: (1) that the exact 1-sided 95% binomial CI for the NPV lie above 99.5% and (2) that the point estimate of sensitivity was at least 95%. We designed the study to provide at least 80% power for achieving the NPV requirement if the true NPV (as per the derivation studies) was at least 99.8% and to provide at least 90% power to achieve the sensitivity requirement if the true sensitivity (as per the derivation studies) was at least 98%. Exact binomial test power calculations using SAS statistical software (SAS Institute) indicated that a sample of at least 2360 patients with rule negative results provided at least 80% power for the NPV requirement and that a sample of 55 patients with the outcomes of interest provided at least 90% power to satisfy the sensitivity requirement.

In the derivation studies, 25.6% of children with IAIs had IAI undergoing acute intervention (primary outcome) and 54.3% with TBIs on CT had clinically important TBI (primary outcome) [7,9]. Annually, each of the participating sites provide care for approximately 208 eligible patients per year with IAIs, 112 eligible patients aged 2-18 years with TBIs on CT, and 44 eligible patients aged <2 years with TBIs on CT. With these rates and an estimated enrollment rate of 80% of eligible patients, we anticipated enrolling 128 patients with IAIs undergoing acute intervention, 146 patients aged 2-18 years with clinically important TBIs, and 57 patients aged <2 years with clinically important TBIs. Thus, we anticipated meeting the sample size for the study’s sensitivity requirements.

Therefore, we ultimately anticipated enrolling more than 20,000 children with blunt head trauma and 7500 children with blunt abdominal trauma. Since at least 20% of children with head trauma also have abdominal trauma [9], we anticipated enrolling nearly 24,000 patients (including patients with isolated head, isolated abdomen, or both head and abdominal trauma) to meet the sensitivity sample sizes required for the validation of both the IAI and TBI prediction rules. Given the above calculations and the expected number of patients with blunt head and abdominal trauma, we estimated approximately 36-39 months of patient enrollment (and budgeted 42 months to ensure meeting sample size requirements). Therefore, with these estimates, the sample would allow a definitive assessment of the validity of the IAI clinical prediction rule and the 2 age-stratified TBI clinical prediction rules.

Based on anticipated enrollment rates to meet the study’s sensitivity requirements, 3200 patients enrolled into the IAI study would be negative for the IAI prediction rule (42% of enrolled patients would be negative for the rule based on prior data) [7]. For the TBI age cohorts, we anticipated enrolling 8600 patients aged 2-18 years with negative results for the TBI rule (59% of enrolled patients would be negative for the rule based on prior data) and 3000 patients aged <2 years would be enrolled who are negative for the prediction rule based on prior data [9]. Thus, we would have more than ample patients to meet the NPV sample size requirements.

#### Interrater Reliability of the Clinical Prediction Rules

The usefulness of ED clinical prediction rules greatly depends on the reliability (reproducibility of findings) of patient history and physical examination variables [27,28], which we had already measured with great precision for the individual predictors in both rules [35,36]. In this study, however, we estimated Cohen κ for each clinical prediction rule as a whole (ie, clinician agreement that the patient is positive or negative for the prediction rule) and used it to assess the null hypothesis that the true κ value was no higher than 0.6. ED clinicians at the participating sites who were not responsible for the care of the patient performed an independent clinical assessment on a convenience sample of eligible patients (within 60 minutes of the first assessment) to fulfill the interrater reliability assessment of the prediction rule. We specified this to be an asymptotic 97.5% 1-sided CI for Cohen κ that lies strictly above 0.6. The clinicians performing this second assessment were unaware of the results of the initial assessment. We specified that a true κ of 0.7 was important to be able to detect with at least 90% power. For the IAI rule, where we anticipated that 55% would be considered rule-positive by each rater, a conservative power calculation using the approach by Cantor [37] indicated that a sample of 680 patients (each assessed by 2 raters) would satisfy the stated requirements (at least 90% power to detect a true κ of 0.7 under 1-sided testing with α=2.5%). Similarly, for the TBI rule, based on our prior study, we anticipated that 42% of the sample would be considered rule-positive, and a sample of 680 patients would satisfy the stated requirements.

### Ethics Approval

This study was conducted in compliance with IRB and HIPAA regulations. The University of California Davis IRB served as the central IRB for the study (920170).

### Patient Consent and Data Security

The study involved minimal risk to patients, families, and participating clinicians. Since the initial enrollment of patients was conducted under a waiver of informed consent, we ensured the protection of the privacy interests of the patients by only discussing with the patient’s treating team when completing the data collection activities in the ED. For those patients where telephone or SMS text message follow-up contact was planned, verbal consent and an information sheet were provided to the guardians in the patient’s ED rooms to ensure that their participation in the study remained private. When conducting any follow-up telephone calls or SMS text messaging, we verified that we were speaking to the correct individual before going into the details of the study. For the SMS text message follow-up portion of the study, we used the StudyPages program (Yuzu Labs PBC), which is a HIPAA-compliant, participant recruitment and engagement platform for clinical research that enables and facilitates secure communication and data transfer between participants and the study team. Clinicians who were eligible for the survey portion of this study were emailed a link to the survey, which allowed them to complete the survey in a location of their choosing, thus protecting their privacy.

## Results

The project was funded in 2016. Patient enrollment began on December 28, 2016, and was completed on September 1, 2021. Patient enrollment was extended due to the COVID-19 pandemic. The COVID-19 pandemic resulted in an initial halt to all non–COVID-19 research activities at participating study sites, after which study enrollment resumed until completion. As of September 1, 2021, over 7500 patients were enrolled in the IAI cohort, over 20,000 patients were enrolled in the TBI cohort, and over 4700 patients were enrolled in both. Additionally, over 450 clinicians were enrolled in the clinician survey. Data clean-up and analyses are projected to be completed by December 2022, and the results are expected to be submitted for peer-reviewed publication in 2023.

## Discussion

We expect this large, multicenter prospective cohort study to successfully validate previously derived, highly sensitive, and specific prediction rules for the evaluation of children in the ED following blunt abdominal or head trauma. With the completion of this study, and assuming successful validation, these prediction rules can undergo widespread implementation. Clinician use of these rules will improve care and encourage scientifically based clinical decision-making in pediatric trauma care. Potential reductions in the frequency of CT scanning will not only lead to safer care but also substantial cost savings. These validated rules could also increase efficiency in care by decreasing false positive results and costs associated with the care of future malignancies in those inappropriately imaged with CT.

The evaluation and treatment of patients in the ED is prone to inefficiencies and errors. This is in great part due to the clinical uncertainties of rapidly caring for patients who are acutely injured and the limited patient history and clinical data available to clinicians practicing in the ED setting. Clinical prediction rules help ED clinicians reduce the diagnostic and therapeutic uncertainties inherent to that setting [28]. The validation of these rules in a large, diverse population will provide clinicians with the definitive evidence to accurately and safely use CT scans in children at risk of substantial injuries while avoiding unnecessary CT scans [26]. This will decrease both the rates of errors of omission and commission. The validation and implementation of these prediction rules will improve child health outcomes through the support of evidence-driven practice for the evaluation of children who are injured.

The IAI prediction rule has not been previously validated in a prospective study. Several retrospective validations of the IAI rule exist, suggesting a prospective validation is likely to be successful [38-40]. Several prospective studies validating the PECARN TBI rules have been performed [41-46]. These studies that vary widely in sample size have indicated that the TBI rules consistently perform accurately.

The strengths of our study include the large, multicenter cohort from a geographically diverse set of EDs and rigorous methodology applied to the study design. The limitations of our study include that CT scans were not obtained for all children. However, we followed patients after their visits to ascertain any missed injuries. Additionally, we did not include community or rural EDs; however, previous research has shown applicability in these other settings [47,48].

Overall, this prospective cohort study was designed to validate previously derived prediction rules for the evaluation of children in the ED following blunt abdominal or head trauma. With implementation, this will result in improved care delivery for children who are injured. Future studies should focus on impact analyses of the prediction rules once successfully implemented.

## Appendix

Multimedia Appendix 1 Emergency department case report data collection form.

## Abbreviations

CT: computed tomography
ED: emergency department
HIPAA: Health Insurance Portability and Accountability Act
IAI: intra-abdominal injury
IRB: institutional review board
NPV: negative predictive value
PECARN: Pediatric Emergency Care Applied Research Network
REDCap: Research Electronic Data Capture
TBI: traumatic brain injury

## Data Availability

All data obtained during the study will be placed in a database for future use after being stripped of identifiers. Deidentified data will be reposited within the Eunice Kennedy Shriver National Institute of Child Health and Human Development (NICHD) Data and Specimen Hub, a centralized resource for researchers to store deidentified data and to access data and associated biospecimens from NICHD-supported studies for use in secondary research [49].
