# Supplementary material for: Validation of Prediction Rules for Computed Tomography Use in Children With Blunt Abdominal or Blunt Head Trauma: Protocol for a Prospective Multicenter Observational Cohort Study
Source: JMIR Res Protoc. 2022 Nov 24;11(11):e43027. doi: 10.2196/43027 (PMC9732756; doi:10.2196/43027)
Supplement: Multimedia Appendix 1 [file resprot_v11i11e43027_app1.pdf]

IAI and TBI Decision Rule Validation Study: ED Data Collection Form #1

Complete this form: **Prior to reviewing CT (if obtained)**
Data Source: faculty, fellow, NP, PA, or resident physician with faculty/fellow oversight

Patient Sticker Here:

Patient name #:____________________________

MR #:_____________________________________

Name of faculty or fellow physician responsible for this patient

____

******COMPLETE SECTIONS I AND II FOR ALL PATIENTS******

I. Enrollment criteria: Abdominal sub-study (Age < 18 years)

A. Inclusion Criteria (Must be "Yes" to at least one inclusion criteria to qualify for the study):

☐ Yes ☐ No….. Blunt torso trauma resulting from a significant mechanism of injury (Motor vehicle collision: greater than 60mph, ejection, or rollover, Automobile versus pedestrian/bicycle: automobile speed greater than 25mph, Falls greater than 20 feet in height, Crush injury to the torso, Physical assault involving the abdomen)
☐ Yes ☐ No….. Decreased level of consciousness (GCS score < 15) in association with blunt torso trauma
☐ Yes ☐ No….. Blunt traumatic event with either of the following (regardless of the mechanism): extremity paralysis or multiple long bone fractures at multiple sites (e.g., tibia and humerus fracture)
☐ Yes ☐ No….. History and physical examination suggestive of IAI following blunt torso trauma: This would include any abdominal imaging (CT or FAST), chest and pelvis x-rays, or laboratory screening for IAI.

- **If any are marked YES, move to section B) Exclusion Criteria**

B. Exclusion Criteria IAI sub-study (any of the below)

☐ Yes ☐ No….Penetrating trauma (e.g. gunshot wound and knife)
☐ Yes ☐ No….Pre-existing neurological disorders seriously complicating physical examination assessment
☐ Yes ☐ No….Traumatic injury occurred more than 24 hours ago
☐ Yes ☐ No….Transfer of the patient with prior abdominal CT
☐ Yes ☐ No….Strong suspicion that the injury is the result of child abuse (E.g. plan to obtain a skeletal survey).
☐ Yes ☐ No….Known pregnancy.

If ALL exclusions are NO, the patient IS eligible for the IAI sub-study, please complete pages 2, 3 and 4 and determine TBI eligibility below

II. Enrollment criteria: Head sub-study (Age < 18 years)

A. Inclusion Criteria (Must be "Yes" to at least one inclusion criteria to qualify for the study):

☐ Yes ☐ No…..Children <18 years with non-trivial blunt head trauma within 24 hours of injury
☐ Yes ☐ No…..A head CT is being performed following trauma

- **If any are marked YES, move to section B) Exclusion Criteria**

B. Exclusion Criteria TBI sub-study (any of the below)

☐ Yes ☐ No….Trivial head trauma: the mechanism of injury is a fall from ground level, or walking or running into stationary objects, *and* the patient lacks any signs or symptoms of head trauma or the presence of scalp lacerations or abrasions alone
☐ Yes ☐ No….Penetrating trauma (e.g. gunshot wound and knife)
☐ Yes ☐ No….Pre-existing neurological disorders seriously complicating physical examination assessment
☐ Yes ☐ No….Pre-existing VP or VA shunts (or similar devices)
☐ Yes ☐ No….Traumatic injury occurred more than 24 hours ago
☐ Yes ☐ No….Transfer of the patient with prior cranial CT/MRI
☐ Yes ☐ No….Pre-existing brain tumor or bleeding disorder
☐ Yes ☐ No….Strong suspicion that the injury is the result of child abuse (E.g. plan to obtain a skeletal survey).
☐ Yes ☐ No….Known pregnancy.

If ALL exclusions are NO, the patient IS eligible for the Head sub-study, please complete pages 2, 5, and 6.

Please complete data sheet prior to reviewing any CT (if CT is obtained)

PLEASE PLACE COMPLETED FORMS IN THE LOCKED DATA COLLECTION BOX

REMEMBER TO PROVIDE GUARDIAN WITH INFORMATION SHEET.

☐-☐☐☐☐

Site # Patient #

1. GENERAL INFORMATION

Completed by ED clinician

Race (Clinician assessment, check all that apply)

☐ American Indian or Alaskan Native	☐ Asian	☐ Black or African American
☐ Native Hawaiian or Other Pacific Islander	☐ White	☐ Unknown
☐ Other		

Ethnicity Clinician assessment

☐ Non-Hispanic and Non-Latino	☐ Hispanic or Latino	☐ Unknown

Mechanism of Injury (check one)

☐ Motor vehicle collision
☐ Fall down stairs
☐ Pedestrian struck by moving vehicle
☐ Motorcycle/ATV/motorized scooter collision
☐ Object struck abdomen
☐ Unknown
☐ Fall from elevation
☐ Ground level fall/Ran into a stationary object
☐ Bicyclist struck by moving vehicle or fall off bike
☐ Assault
☐ Object struck head
☐ Other (list): ______________________________

Initial Glasgow Coma Scale (GCS) score: ______

Completed by ED Clinician, RC, or other research staff

**Date and Estimated Time of Injury (24 hour clock, midnight = 00:00)** ☐ Time of injury is unknown	**Date and Time of ED Evaluation (24 hour clock, midnight = 00:00)**
______/______/________ ________:_____	______/______/________ ________:_____
(mm) (dd) (yyyy) (hh) (mm)	(mm) (dd) (yyyy) (hh) (mm)

Was the parent/guardian available (in the room) for questioning during the INITIAL evaluation of the injured child?

☐ No (answer questions below) ☐ Yes (proceed with remainder of the data sheet)

If the parent/guardian was not available, the reason the parent/guardian was not available was:
(check all that apply)

☐ they were also involved in the traumatic event and are at a different hospital
☐ they were also involved in the traumatic event and are currently being treated here
☐ they were also involved in the traumatic event but we are unaware where they are or paramedics state that the parents are "on their way"
☐ they did not ride in the ambulance that transported the patient
☐ child was brought to the ED by someone other than his or her parent/guardian
☐ it is unclear where they were at the time of initial evaluation of the child
☐ other ____

Approximate time of parent/guardian arrival (24hr time): _____:_____ (hh) (mm) **Date:** _____/_____/_____ (mm) (dd) (yyyy) *(If different than date of patient presentation)*

☐ Time of parent/guardian arrival is unknown
☐ Parent/guardian never arrived while patient was in the ED

**Name of Parent/Guardian/Responsible Family Member:**	
**Number/pager to Reach Guardian/Parent** (______) ______ -- ________________ **Preference:** ☐Call ☐Text ☐No preference	**Alternate Number for Guardian/Parent** (______) ______ -- ________________ **Preference:** ☐Call ☐Text ☐No preference
**Parent/Guardian/Responsible Family Member principal language:** ☐ English ☐ Spanish* ☐ Chinese*	☐ Russian* ☐ Hmong* ☐ Other*____________________
***If primary language is not English, does the parent/ guardian/ responsible family member also speak English?**	☐ Yes ☐ No

Was letter of information and HIPAA form given to patient's *guardian*? ☐Yes ☐No

Check here if the patient's *guardian* _refused_ *telephone follow-up call*: ☐

IAI

Complete Abdominal Injury Questions only if the Patient is Eligible for Abdominal Trauma Sub-study

2. Abdominal Injury Decision Rule Variables

a) **Is the patient positive for any Abdomen Prediction Rule variable (i.e. "Yes" to any below):** ☐ Yes ☐ No
If no, go to question b below, if yes, answer questions in box:

**Does the patient complain of abdominal pain?**	☐ Yes	☐ No	☐ Unknown
**Has the patient vomited since the time of injury?**	☐ Yes	☐ No	☐ Unknown
**Is the patient's GCS score < 14?**	☐ Yes	☐ No	☐ Unknown
**Does the patient have absent/decreased breath sounds?**	☐ Yes	☐ No	☐ Unknown
**Does the patient have any thoracic wall trauma?** (erythema, abrasions, ecchymosis, subcutaneous air, laceration, etc)	☐ Yes	☐ No	☐ Unknown
**Does the patient have any abdominal wall trauma?** (seat belt sign, erythema, abrasions, ecchymosis, subcutaneous air, laceration, etc)	☐ Yes	☐ No	☐ Unknown
**Does the patient have abdominal tenderness?**	☐ Yes	☐ No	☐ Unknown

Suspicion of Injury (immediately after history and exam but prior to ancillary testing, ultrasound):

b) What is your suspicion that this patient has an intra-abdominal injury?
☐ **<1%** ☐ **1-5%** ☐ **6-10%** ☐ **11-50%** ☐ **>50%**

c) What is your suspicion that this patient has an intra-abdominal injury that will undergo therapy (therapeutic laparotomy for an IAI, blood transfusion for abdominal hemorrhage, IV fluids for 2 or more nights in patients with pancreas/GI injuries) or lead to death from the IAI?
☐ **<1%** ☐ **1-5%** ☐ **6-10%** ☐ **11-50%** ☐ **>50%**

d) What were the most important factors you used for obtaining an abdominal CT?
→ check all that apply

☐ Young age	☐ FAST examination abnormal
☐ Severe mechanism of injury	☐ Abdominal clearance prior to non-abdominal surgery
☐ Lower rib injury	☐ Low or declining hematocrit
☐ Hemodynamic instability	☐ Elevated AST or ALT
☐ Decreased Mental status	☐ Elevated amylase/lipase
☐ Flank tenderness	☐ Microscopic hematuria
☐ Femur fracture	☐ Gross hematuria
☐ Abnormal abdominal examination	☐ Other abnormal lab value (describe) ___________________________
☐ Trauma Surgery Request	☐ Other (describe) ____
☐ Parental request	☐ N/A: No abdominal CT ordered

e) Was a FAST (abdominal ultrasound) performed in the ED? ☐ Yes ☐ No
If yes, was intraperitoneal fluid/free fluid identified (FAST positive)? ☐ Yes ☐ No ☐ Indeterminate

f) Was the patient observed prior to the decision to obtain or not obtain an Abdominal CT?
☐ Yes → Complete Questions f1, f2 and f3 ☐ No → go to next page for TBI study

f1) Please check all the reasons that you decided to observe prior to deciding whether or not to obtain an abdominal CT (choose all that apply)

- ☐ Severe mechanism of injury
- ☐ Abdominal pain
- ☐ Abdominal tenderness
- ☐ GCS < 15
- ☐ Abdominal wall trauma
- ☐ Vomiting
- ☐ Abnormal breath sounds
- ☐ Costal margin tenderness
- ☐ Chest wall trauma
- ☐ Flank tenderness
- ☐ Other___________________________________

f2) Prior to decision to obtain a CT or not, the patient's abdominal tenderness:

- ☐ Pt never had abdominal tenderness
- ☐ Improved (but did not resolve)
- ☐ Stayed the same
- ☐ Can't assess – Pt preverbal or nonverbal
- ☐ Worsened
- ☐ Did not reassess for abdominal tenderness
- ☐ Resolved with analgesia
- ☐ Resolved without analgesia

f3) Prior to decision to obtain a CT or not, the patient's vomiting:

- ☐ Pt never vomited
- ☐ Stayed the same (vomiting continued)
- ☐ Resolved without meds
- ☐ Worsened
- ☐ Resolved with meds
- ☐ Did not reassess for vomiting

Continue to next page for Head Decision Rules

☐-☐☐☐☐
Site # Patient #

TBI

Complete Head Injury Questions only if the Patient is Eligible for Head Trauma Sub-study

3. Did the patient have a post-traumatic seizure? ☐ **Yes** ☐ **No** ☐ **Unknown**

4. Is the patient's GCS score <14? ☐ **Yes** → **Stop, not eligible for head trauma decision rule study below**
☐ **No** → **Complete appropriate information below if patient has head trauma**

AGE < 2 years

5. Head Injury Prediction Rule Variables AGE < 2 years (less than 24 months)

a) Is the patient positive for any TBI Prediction Rule variables (i.e. "Yes" to any of the below):
☐ **Yes** ☐ **No**
If yes, answer all the questions in box:

**Does the patient have altered mental status or GCS<15?** (slow to respond, agitation, sleepiness, confusion, or repetitive questioning)	☐ Yes	☐ No	☐ Unknown
**Does the patient have a non-frontal scalp hematoma?**	☐ Yes	☐ No	☐ Unknown
**Was there a loss of consciousness ≥ 5 seconds?**	☐ Yes	☐ No	☐ Unknown
**Does the patient have a palpable skull fracture or is unclear due to scalp swelling?**	☐ Yes	☐ No	☐ Unknown
**Is the patient acting abnormally per the parent/guardian?**	☐ Yes	☐ No	☐ Unknown
**Was there a severe mechanism of injury?**	☐ Yes	☐ No	☐ Unknown

Severe mechanism of injury includes motor vehicle crash with patient ejection, death of another passenger, or rollover; pedestrian or bicyclist without helmet struck by a motorized vehicle; fall > 3 feet; or head struck by a high-impact object (significantly heavily object struck head, baseball, horse kick, etc)

OR AGE ≥ 2 years

5. Head Injury Prediction Rule Variables AGE ≥ 2 years (24 months or older)

a) Is the patient positive for any TBI Prediction Rule variables (i.e. "Yes" to any of the below):
☐ **Yes** ☐ **No**
If yes, answer all the questions in box

**Does the patient have altered mental status or GCS<15?** (slow to respond, agitation, sleepiness, confusion, or repetitive questioning)	☐ Yes	☐ No	☐ Unknown
**Did the patient have a loss of consciousness?**	☐ Yes	☐ No	☐ Unknown
**Has the patient vomited since the injury?**	☐ Yes	☐ No	☐ Unknown
**Are there clinical signs of basilar skull fracture?**	☐ Yes	☐ No	☐ Unknown
**Does the patient have a severe headache (scale 8 – 10)?**	☐ Yes	☐ No	☐ Unknown
**Was there a severe mechanism of injury?**	☐ Yes	☐ No	☐ Unknown

Severe mechanism of injury includes motor vehicle crash with patient ejection, death of another passenger, or rollover; pedestrian or bicyclist without helmet struck by a motorized vehicle; fall >5 feet; or head struck by a high-impact object (significantly heavily object struck head, baseball, horse kick, etc)

Suspicion of Injury (immediately after history and exam):

b) What is your suspicion that this patient will have a TBI identified on CT (regardless of whether a CT is being obtained)?

☐ **<1%** ☐ **1-5%** ☐ **6-10%** ☐ **11-50%** ☐ **>50%**

c) What is your suspicion that this patient has a clinically important TBI (death from the TBI, neurosurgery for TBI, intubation >24 hours for TBI, or injury visualized on CT scan in association with hospitalization ≥2 nights for the head trauma)?

☐ **<1%** ☐ **1-5%** ☐ **6-10%** ☐ **11-50%** ☐ **>50%**

**d) What were the most important factors you used for obtaining a cranial CT?** → check all that apply	
☐ Young age	☐ Decreased mental status
☐ Severe mechanism of injury	☐ Clinical evidence of skull fracture
☐ LOC	☐ Scalp hematoma
☐ Amnesia	☐ Neurologic deficit (other than mental status)
☐ Seizure	☐ Parental request
☐ Headache	☐ Trauma Surgery Request
☐ Skull fracture on x-ray	☐ Other (describe) ________________________________
☐ Referring MD request	☐ N/A: No cranial CT ordered
☐ Vomiting	

e) Was the patient observed prior to the decision to obtain or not obtain a Head CT?

☐ Yes → Complete Questions e1, e2, e3 and e4 ☐ No → STOP, form is complete

e1) Please check all the reasons that you decided to observe prior to deciding whether or not to obtain a head CT:

☐ Severe mechanism of injury
☐ Scalp trauma
☐ Headache
☐ GCS < 15 or altered mental status
☐ History of loss of consciousness/amnesia
☐ Vomiting
☐ Nausea
☐ Patient acting abnormally per parents
☐ Seizure
☐ Other________________________

e2) Prior to CT or if no CT obtained, the patient's headache:

☐ Pt never had headache
☐ Stayed the same
☐ Resolved with analgesia
☐ Improved (but did not resolve)
☐ Worsened
☐ Resolved without analgesia
☐ Can't assess – Pt preverbal or nonverbal
☐ Did not reassess

e3) Prior to CT or if no CT obtained, the patient's vomiting:

☐ Pt never vomited
☐ Worsened
☐ Stayed the same
☐ Resolved with meds
☐ Resolved without meds
☐ Did not reassess

e4) Prior to CT or if no CT obtained, the patient's mental status/GCS:

☐ Pt never had GCS <15
☐ Did not reassess
☐ Improved to normal (GCS 15)
☐ Improved but not to normal
☐ Stayed the same
☐ Worsened
